# Current and future distribution of *Aedes aegypti* and *Aedes albopictus* (Diptera: Culicidae) in WHO Eastern Mediterranean Region

**DOI:** 10.1186/s12942-018-0125-0

**Published:** 2018-02-14

**Authors:** Els Ducheyne, Nhu Nguyen Tran Minh, Nabil Haddad, Ward Bryssinckx, Evans Buliva, Frédéric Simard, Mamunur Rahman Malik, Johannes Charlier, Valérie De Waele, Osama Mahmoud, Muhammad Mukhtar, Ali Bouattour, Abdulhafid Hussain, Guy Hendrickx, David Roiz

**Affiliations:** 1 Avia-GIS, Zoersel, Belgium; 2 Regional Office for the Eastern Mediterranean, World Health Organisation, Cairo, Egypt; 3 Laboratory of Immunology and Vector-Borne Diseases, Faculty of Public Health, Lebanese University, Fanar, Lebanon; 4 MIVEGEC Lab, IRD/CNRS/UM, Montpellier, France; 5 Directorate General for Disease Surveillance and Control, Ministry of Health, Muscat, Sultanate of Oman; 6 Directorate of Malaria Control, Islamabad, Pakistan; 7 Institut Pasteur Tunis, Tunis, Tunisia; 8 Vector Control Focal Point, Ministry of Health, Puntland, Somalia

**Keywords:** *Aedes*, *Aedes aegypti*, *Aedes albopictu*s, Distribution, Chikungunya, Dengue, Spatial model, Surveillance, Yellow fever, Zika

## Abstract

**Background:**

*Aedes*-borne diseases as dengue, zika, chikungunya and yellow fever are an emerging problem worldwide, being transmitted by *Aedes aegypti* and *Aedes albopictus*. Lack of up to date information about the distribution of *Aedes* species hampers surveillance and control. Global databases have been compiled but these did not capture data in the WHO Eastern Mediterranean Region (EMR), and any models built using these datasets fail to identify highly suitable areas where one or both species may occur. The first objective of this study was therefore to update the existing *Ae. aegypti* (Linnaeus, 1762) and *Ae. albopictus* (Skuse, 1895) compendia and the second objective was to generate species distribution models targeted to the EMR. A final objective was to engage the WHO points of contacts within the region to provide feedback and hence validate all model outputs.

**Methods:**

The *Ae. aegypti* and *Ae. albopictus* compendia provided by Kraemer et al. (Sci Data 2:150035, 2015; Dryad Digit Repos, 2015) were used as starting points. These datasets were extended with more recent species and disease data. In the next step, these sets were filtered using the Köppen–Geiger classification and the Mahalanobis distance. The occurrence data were supplemented with pseudo-absence data as input to Random Forests. The resulting suitability and maximum risk of establishment maps were combined into hard-classified maps per country for expert validation.

**Results:**

The EMR datasets consisted of 1995 presence locations for *Ae. aegypti* and 2868 presence locations for *Ae. albopictus*. The resulting suitability maps indicated that there exist areas with high suitability and/or maximum risk of establishment for these disease vectors in contrast with previous model output. Precipitation and host availability, expressed as population density and night-time lights, were the most important variables for *Ae. aegypti*. Host availability was the most important predictor in case of *Ae. albopictus*. Internal validation was assessed geographically. External validation showed high agreement between the predicted maps and the experts’ extensive knowledge of the terrain.

**Conclusion:**

Maps of distribution and maximum risk of establishment were created for *Ae. aegypti* and *Ae. albopictus* for the WHO EMR. These region-specific maps highlighted data gaps and these gaps will be filled using targeted monitoring and surveillance. This will increase the awareness and preparedness of the different countries for *Aedes* borne diseases.

**Electronic supplementary material:**

The online version of this article (10.1186/s12942-018-0125-0) contains supplementary material, which is available to authorized users.

## Background

**Fig. 1:**
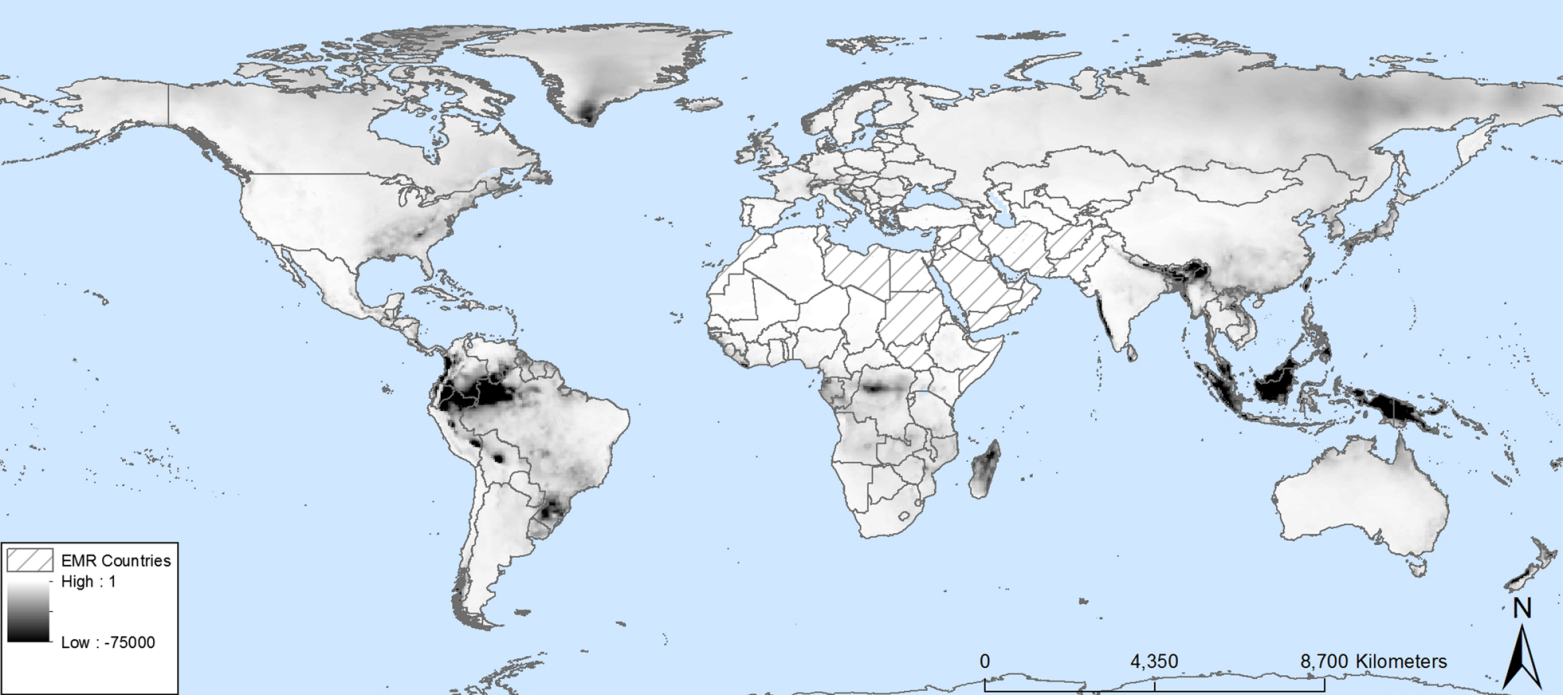
Mahalanobis distance values showing the degree of similarity in environmental conditions between locations worldwide and the EMR

*Aedes*-borne diseases (dengue, chikungunya, yellow fever and zika) are an emerging problem worldwide, escalating overall risk and burden of disease worldwide [3]. Lack of up to date and more precise *Aedes* distributional data and potential distributional modelling hampers effective vector surveillance and control. This is particularly true in the WHO Eastern Mediterranean Region (EMR), a region which includes Afghanistan, Bahrain, Djibouti, Egypt, Iraq, Iran, Jordan, Kuwait, Lebanon, Libya, Morocco, Oman, Pakistan, Palestine, Qatar, Saudi Arabia, Somalia, Sudan, Syria, Tunisia, United Arab Emirates and Yemen (Fig. 1). Detailed information about disease burden and the current and potential spatial distribution of *Aedes* vectors in the EMR is scarce. Humphrey et al. [4] listed the available information about dengue incidence and country scale vector distribution in the region.

Therefore, there is a clear need to focus on this area, especially in the light of the recent zika virus (ZIKV) pandemic. Even though none of the EMR countries had any reported ZIKV transmission, the risk of autochthonous zika transmission in the EMR, especially on the Red Sea rim and Pakistan, following introduction from endemic countries, cannot be overlooked.

Given the wide spread distribution and the abundance of *Aedes aegypti* and the reported cases of dengue, chikungunya and yellow fever, Tran Minh et al. [5] assumed that the potential risk of disease outbreaks is high in at least eight of the EMR countries: Djibouti, Egypt, Oman, Pakistan, Saudi Arabia, Somalia, Sudan and Yemen. Furthermore, in recent years the invasive vector *Aedes albopictus* has spread in some countries, such as Lebanon [6] and Morocco [7], but the available occurrence database is not updated. The first objective of this study is therefore to build an up to date comprehensive dataset of observed presences of both *Aedes aegypti* (Linnaeus, 1762) and *Aedes albopictus* (Skuse, 1895) for the EMR.

Existing spatial distribution models for the two *Aedes* species can currently only be extracted from global modelling outputs [8–10]. In these models, observed presence data are strongly biased in favour of the Americas, the Indian subcontinent, South-East Asia and Europe, and the predicted probability for the EMR may therefore not reflect the actual situation. This bias is supported by the case of the TigerMaps models [11]. These models were based only on Mediterranean data, indicating a higher risk of occurrence of *Ae. albopictus* in Northern Africa as compared to any of the previously-mentioned global models. The second objective in this study is therefore to produce a new set of habitat suitability maps for *Ae. aegypti* and *Ae. albopictus* focused on the EMR. These maps will depict areas of potential introduction and maximum risk of establishment. Kraemer et al. [1, 2] compiled a global database of observed occurrences of *Ae. aegypti* and *Ae. albopictus*. These models improve our knowledge of the species’ distribution globally and provide insight into environmental dependencies. On the other hand, model output can be biased when target area environmental conditions are not properly captured in the training set. Given that in Kraemer’s data less than 1% of the observed points fall geographically within the EMR, a similarity mask was created to include occurrence data only from areas similar in environmental conditions to the EMR.

## Methods

### Study area

The EMR has 22 member states/territories: Afghanistan, Bahrain, Djibouti, Egypt, Iraq, Iran, Jordan, Kuwait, Lebanon, Libya, Morocco, Oman, Pakistan, Palestine, Qatar, Saudi Arabia, Somalia, Sudan, Syria, Tunisia, United Arab Emirates and Yemen (Fig. 1). Many countries in the EMR are particularly vulnerable to communicable disease epidemics, because they are experiencing numerous environmental and social stresses, including armed conflicts, water scarcity, food insecurity, rapid population growth and urbanization. Therefore, these countries often have weak governing institutions and health systems [12], although there does exist a large variability in the EMR.

### Training data

First, the Köppen–Geiger classes [13] per EMR country were determined. A binary classification resulted in a mask where value 1 represents all areas globally with one of the classes found in the EMR countries and value 0 where the condition was not met. Secondly, the Mahalanobis distance was calculated using the set of environmental predictors listed in Table 1. The Mahalanobis distance is a unit-less and scale invariant similarity measure. Its value will increase when the environmental conditions become more and more different than those observed in the target area.

**Table 1:** Overview of the environmental and eco-climatic predictor variables used in spatial distribution modelling of *Ae. aegypti* and *Ae. albopictus* in the EMR

Data layer	Description	Resolution (km)	Units	Origin
Altitude	Elevation above sea level	5 × 5	m	WorldClim^a^
BIO1	Annual mean temperature	5 × 5	°C	WorldClim
BIO2	Mean diurnal range (mean of monthly (max temp − min temp))	5 × 5	°C	WorldClim
BIO3	Isothermality (BIO2/BIO7) (× 100)	5 × 5	%	WorldClim
BIO4	Temperature seasonality (standard deviation × 100)	5 × 5	%	WorldClim
BIO5	Max temperature of warmest month	5 × 5	°C	WorldClim
BIO6	Min temperature of coldest month	5 × 5	°C	WorldClim
BIO7	Temperature annual range (BIO5–BIO6)	5 × 5 km	°C	WorldClim
BIO8	Mean temperature of wettest quarter	5 × 5	°C	WorldClim
BIO9	Mean temperature of driest quarter	5 × 5	°C	WorldClim
BIO10	Mean temperature of warmest quarter	5 × 5	°C	WorldClim
BIO11	Mean temperature of coldest quarter	5 × 5	°C	WorldClim
BIO12	Annual precipitation	5 × 5	mm	WorldClim
BIO13	Precipitation of wettest month	5 × 5	mm	WorldClim
BIO14	Precipitation of driest month	5 × 5	mm	WorldClim
BIO15	Precipitation seasonality (coefficient of variation)	5 × 5	%	WorldClim
BIO16	Precipitation of wettest quarter	5 × 5	mm	WorldClim
BIO17	Precipitation of driest quarter	5 × 5	mm	WorldClim
BIO18	Precipitation of warmest quarter	5 × 5	mm	WorldClim
BIO19	Precipitation of coldest quarter	5 × 5	mm	WorldClim
Fourier transforms of T_max_, T_mean_ and T_min_	Amplitudes 1, 2, and 3	50 × 50	°C	EDENext^b^
	Phases 1, 2, and 3	50 × 50	Day of year	EDENext
Fourier transforms of precipitation	Amplitudes 1, 2 and 3	50 × 50	mm	EDENext
	Phases 1, 2, and 3	50 × 50	Day of year	EDENext
Night-time light	Night-time light	5 × 5	Unit-less	DMSP—NASA^c^
Average NDVI	Global annual sum NDVI	5 × 5	Unit-less	LADA^d^
Human population	Population density grid	5 × 5	Persons/pixel	SEDAC^e^

^a^ http://www.worldclim.org/bioclim

^b^ http://www.edenextdata.com

^c^ http://ngdc.noaa.gov/eog/dmsp/downloadV4composites.html

^d^ http://www.fao.org/geonetwork

^e^ http://sedac.ciesin.columbia.edu/data/set/grump-v1-population-density/data-download

The cut off for similarity pixels was set to the 99% percentile of the Mahalanobis distance within the Köppen–Geiger EMR mask. This procedure retained 1351 out of 17,280 entries for *Ae. albopictus* and 1938 out of 13,991 for *Ae. aegypti* (Additional file 2: Fig. S1).

To extend the dataset with data collected specifically in the EMR, additional *Ae. albopictus* presence/absence data from Lebanon were provided by one of the co-authors (N.H.): 186 locations were sampled during summer 2015 for *Ae. albopictus* presence in Lebanon, and the mosquito was found at 73 of them (unpublished data).

In addition, a literature review and personal communications with entomologists and environmental health officers in the EMR provided further presence locations for both *Ae. aegypti* and *Ae. albopictus* and for *Aedes*-borne disease outbreaks (we excluded seroprevalence studies) in the region of interest. This data set is provided as Additional file 1.

The current compiled data sources contain only observed occurrence data of *Ae. aegypti* and *Ae. albopictus*, except for Lebanon where absence data for *Ae. albopictus* was collected. Many modelling techniques require both occurrence and absence data as input data, except for presence-only modelling techniques such as MaxEnt [14]. While these occurrence-only models have proven their value for species distribution modelling in general [15] and *Aedes* modelling more specifically [16, 17], we feel that including simulated absence data, also called pseudo-absence data, would strengthen the model output. This is not only because extra information is added to the training set but also because the range of modelling techniques that can be used is much wider.

A surface range envelope (SRE) presence-only model was used to define the area suitable for both species together. Pseudo-absences were then generated randomly outside this area within the Köppen–Geiger/Mahalanobis EMR mask as a point shapefile without a minimum distance criterion [18]. SRE models were based on the presence training data included in the Mahalanobis distance mask using the BIOMOD package in R [19]. The suitable area was restricted by removing 1.25, 2.5 and 5% of the outer values in each of the environmental predictor variable envelopes. This yielded three different training datasets per species. The number of pseudo-absences that was generated was set equal to the number of presence data to obtain a balanced training dataset and avoid bias towards predicting presence or absence. No pseudo-absences were generated for Lebanon since absence locations were already available. The final absence dataset consisted of the generated pseudo-absences and the balanced subset of absence locations in Lebanon.

### Environmental predictor data

From literature, temperature is a crucial factor limiting the distribution of *Ae. aegypti* and *Ae. albopictus.* Other listed variables include altitude, rainfall as well as land-use and anthropogenic factors. The predictor variable dataset was collated from a variety of sources and included both ground-measured and remotely-sensed data (Table 1).

All variables, except the Fourier transforms, were available at a spatial resolution of 5 × 5 km. The Fourier MODIS images were processed according to Scharlemann et al. [20] over the period of 2001–2012 and were available from the EDENext data archive (http://www.edenextdata.com/). These Fourier variables were downscaled using spatial inverse distance weighted interpolation. The interpolation results were assigned to 5 × 5 km pixels based on the average of the 12 nearest locations in the grid. A 5 × 5 km resolution land mask was then applied to obtain a fine-resolution border between land and water bodies (Additional file 3: Fig. S2).

### Species distribution modelling

The suitability models were generated using Random Forests (RF) [21]. RF is a powerful data mining tool that can model complex interactions between different predictor variables and determine variable importance with great classification accuracy [22]. RF is a mixture of tree predictors that are randomly constructed by bootstrapping from the complete dataset with replacement but having the same distribution as the full dataset. Random forests of 1000 trees were trained using the VECMAP software (http://www.vecmap.com). Six predictor variables were randomly selected at each node to split it into two new branches. Given that the input data sets were balanced, the cut-off value to differentiate between suitable and unsuitable habitats is 0.5.

Variable importance was assessed using the Gini impurity criterion. The smaller the Gini impurity index, the more accurate the classification of the pixel. The Gini impurity index may therefore be very low when nodes are split by variables that are highly correlated with the species’ probability of occurrence. During the training process, random subsets of predictor variables were considered for each split and each time the variable with the lowest Gini impurity index was chosen. To assess variable importance, the mean decrease in the Gini impurity index in each variable as compared the other variables in the model was calculated.

Although individual classification trees in a RF model are grown based on random subsets of training data, all available data was fed into the training process of our random forest model. The estimated habitat suitability for *Ae. aegypti* and *Ae. albopictus* therefore represents overall agreement by the training dataset to classify an area as suitable or unsuitable for the species. Subsets of the training data can, however, reveal extreme cases, i.e., areas where only a part of the training data would classify the area as suitable. These areas are characterized by environmental and anthropogenic conditions that are not ideal for the species but may be deemed sufficient for them to survive. To consider these extreme areas, 100 subsets of the random forest model, each containing 10 trees, were assessed and the maximum value of each pixel was retained.

### Internal and external validation

The standard deviation per pixel of these 100 subsets of the random forest model, each containing 10 trees, was assessed to evaluate model uncertainty, permitting a more geographically based assessment of model uncertainty instead of using overall performance indices. A high pixel value represents large variability in the modelled probabilities of occurrence and therefore greater uncertainty. Pixels with small values indicate that many model repetitions estimated a similar probability of occurrence and therefore represent locations for which the model outcome is more robust.

To externally validate the model outputs, we contacted the WHO EMR point of contacts for every country in the region. To facilitate the interpretation, the four maps per country (current and maximum risk of establishment for *Ae. aegypti* and *Ae. albopictus* respectively) were combined into a single map. This map was obtained by hard classifying the suitable and maximum risk of establishment maps with a threshold of 0.5. This means that if the probability is higher than 0.5 a value of 1 (current) and 2 (maximum risk of establishment) respectively was attributed. In the next step, the maps were combined and the maximum value of each map was retained in the final output.

The experts were asked to assess the model output by indicating areas that are of interest either because they confirm what was found during surveillance activities that were not yet reported or conversely because they show a mismatch between the predicted suitability and the observed suitability. The areas of agreement and disagreement where annotated on the maps and digitized. This was used as input for a confusion matrix with a random sample of 2000 pixels were used to quantify the experts’ opinions, and the following accuracy indices were calculated: Percent Correctly Classified (PCC), Cohen’s index of agreement $\kappa$ and the sensitivity and specificity per class.

## Results

### Training data

**Fig. 2:**
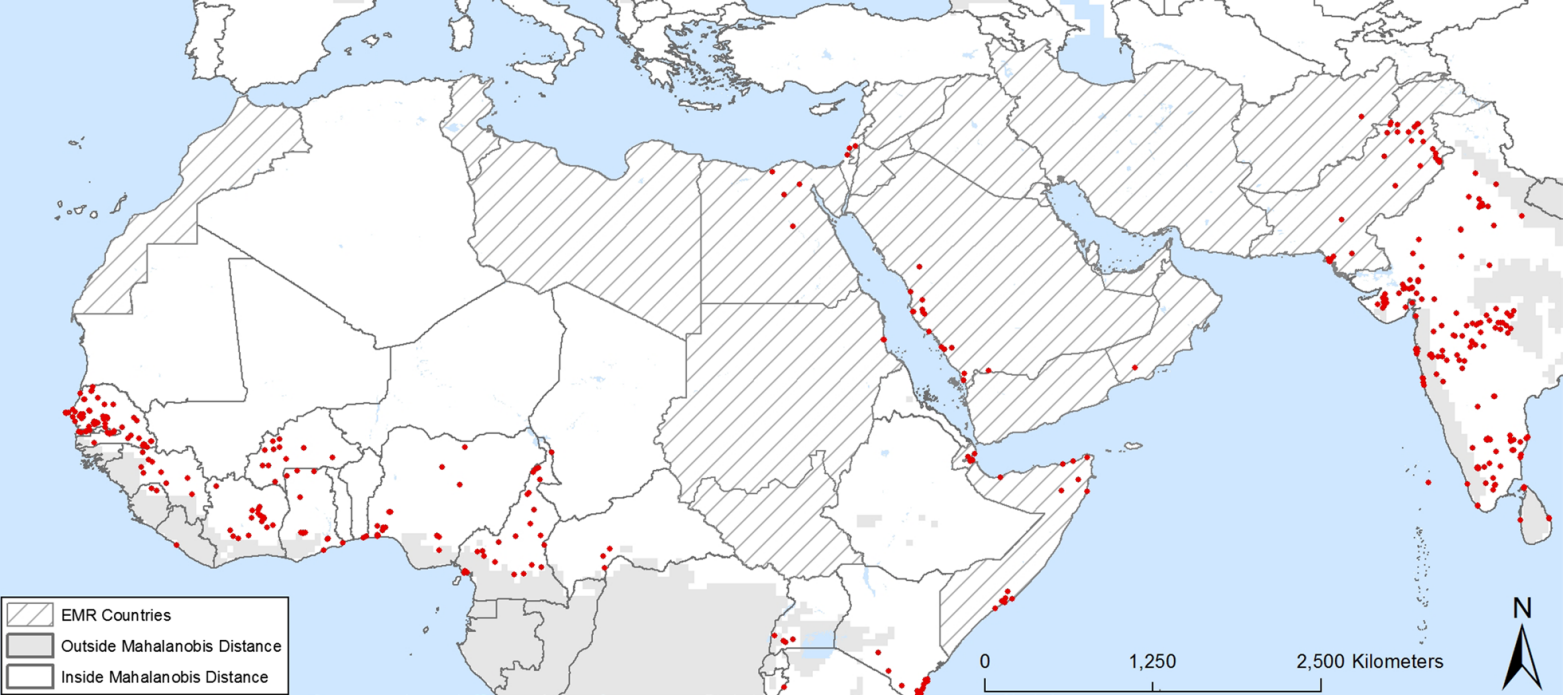
Locations where *Ae. aegypti* was found within the EMR Mahalanobis distance

**Fig. 3:**
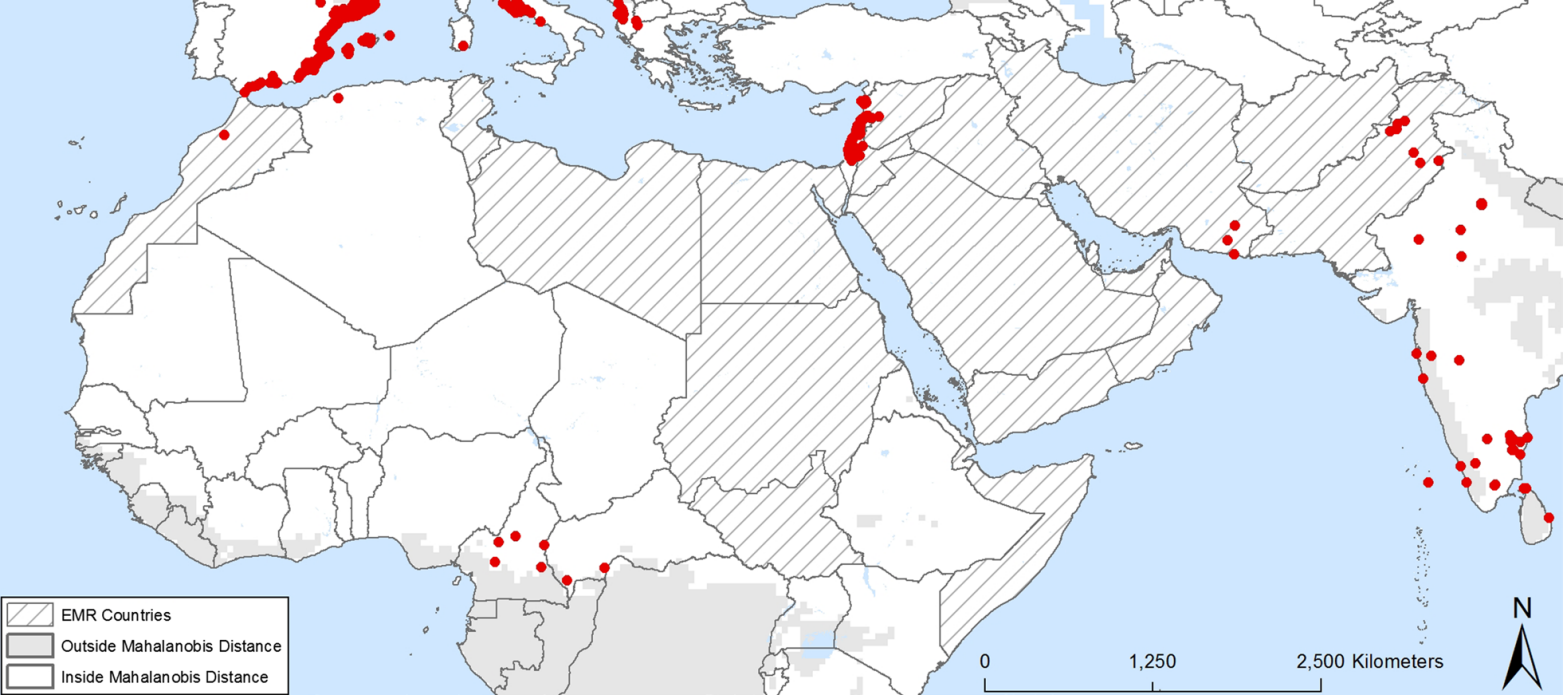
Locations where *Ae. albopictus* was found within the EMR Mahalanobis distance

Following the literature review and the expertise of entomologists in the region, the presence of *Aedes aegypti* was confirmed in several countries of the EMR: Djibouti, Egypt, Oman, Pakistan, Saudi Arabia, Somalia, Sudan, and Yemen (Fig. 2). The presence of *Ae. albopictus* was confirmed in Iran, Jordan, Lebanon, Morocco, Pakistan and Syria (Fig. 3). The two species were detected in nearby regions that are not part of the EMR, such as *Ae. aegypti* in Israel and Turkey and *Ae. albopictus* in Algeria, Israel and Turkey. Dengue was reported in Djibouti, Egypt, Pakistan, Saudi Arabia, Somalia, Sudan and Yemen. Imported dengue cases were reported in Oman and Iran. Chikungunya was reported in Djibouti, Pakistan, Saudi Arabia, Somalia and Yemen, and yellow fever in Sudan. So far, no zika infection was reported in the EMR [3].

Based on this information, the resulting dataset included 1995 locations for *Ae. aegypti* and 2868 presence locations for *Ae. albopictus* within the boundaries of the Mahalanobis distance mask.

### Species distribution modelling

**Fig. 4:**
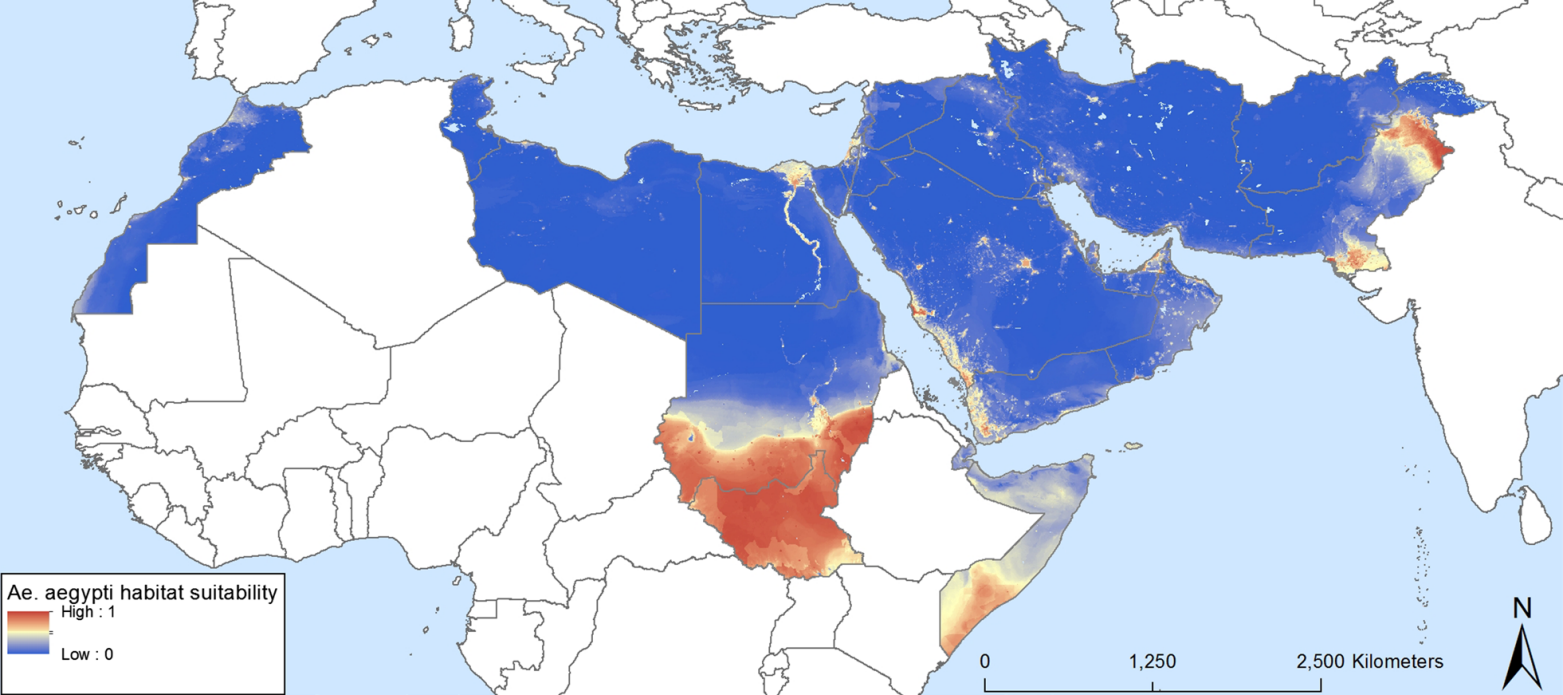
Predicted probability of *Ae. aegypti* occurrence obtained from a random forest model

**Fig. 5:**
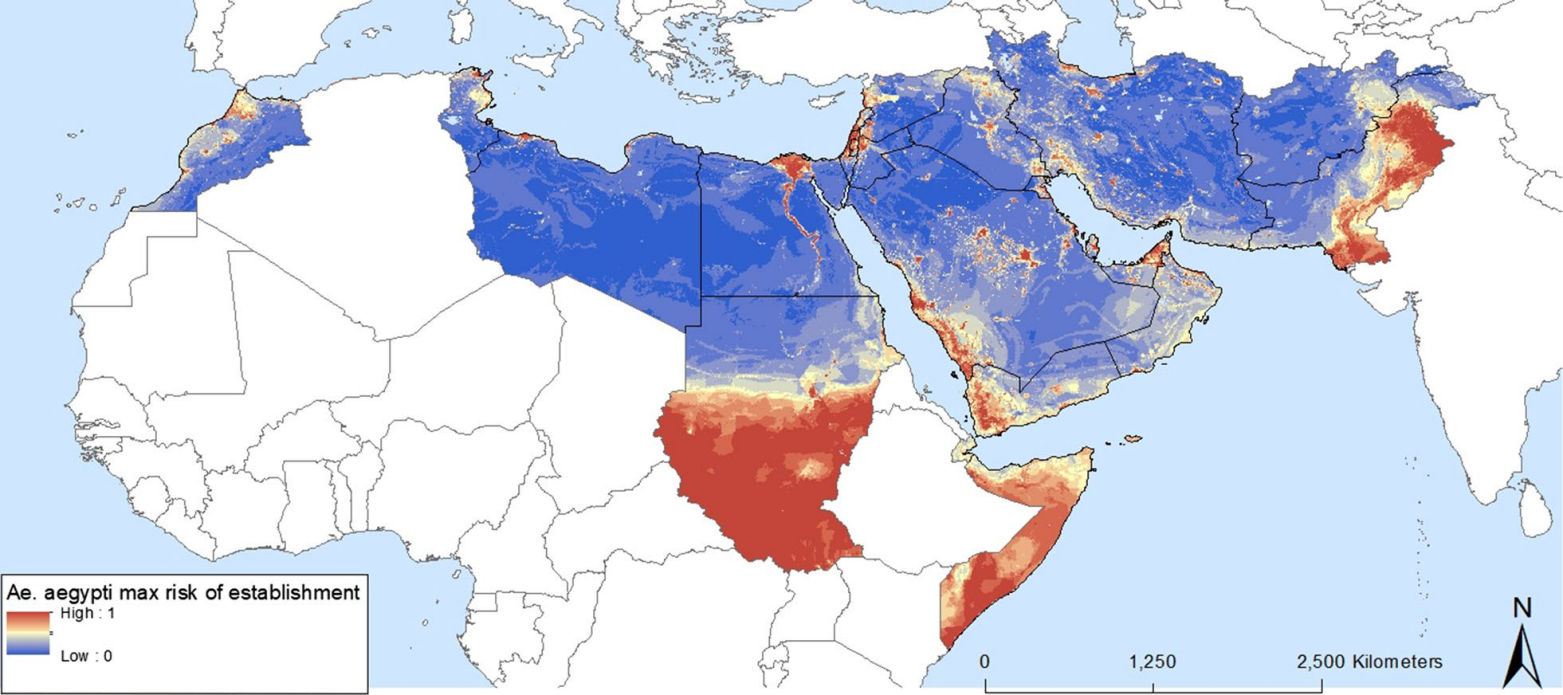
Predicted probability of *Ae. aegypti* occurrence using maximum values at the pixel level from a series of 100 random forest models

**Fig. 6:**
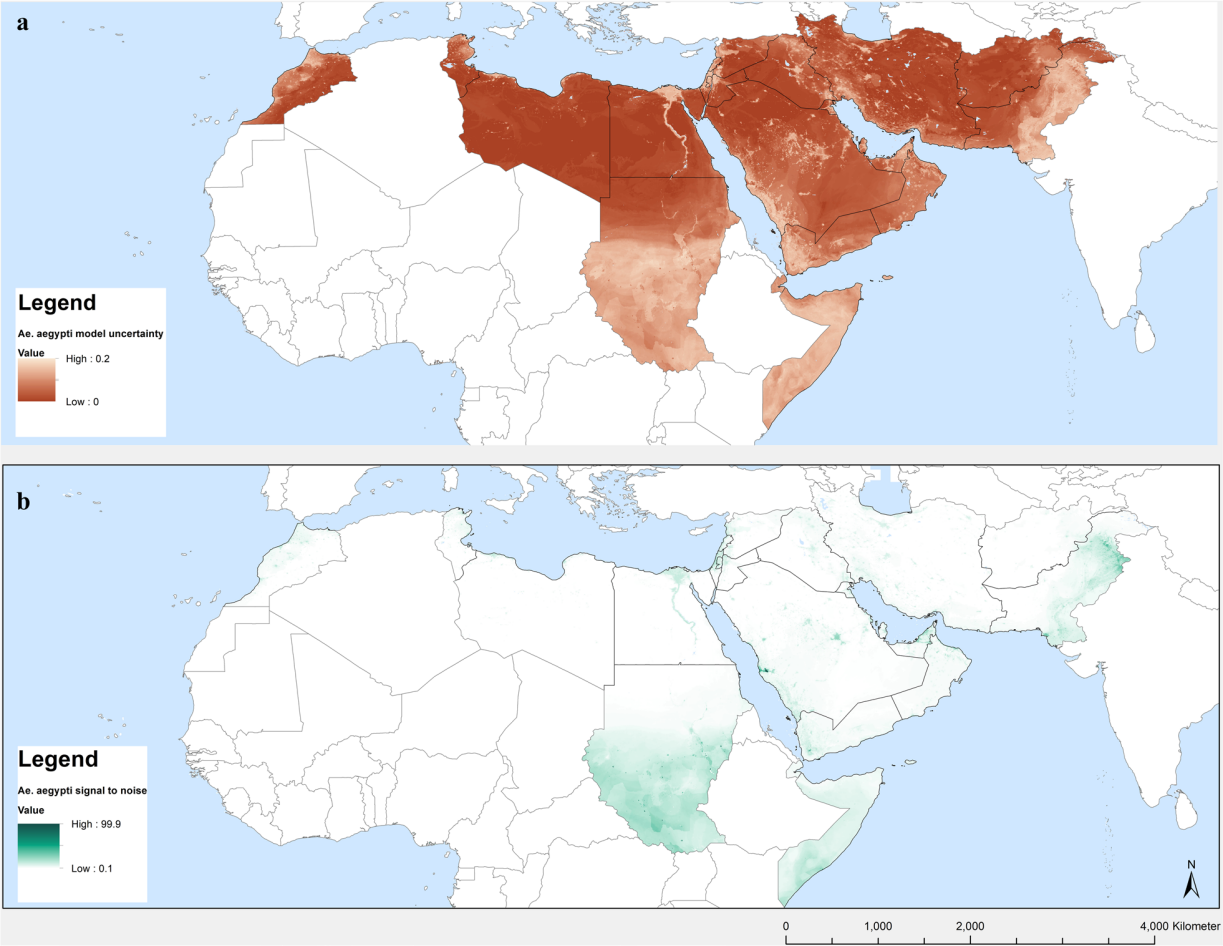
**a** Uncertainty of the *Ae. aegypti* predicted probability of occurrence using standard deviations at the pixel level from a series of 100 random forest models and **b** signal to noise ratio

**Fig. 7:**
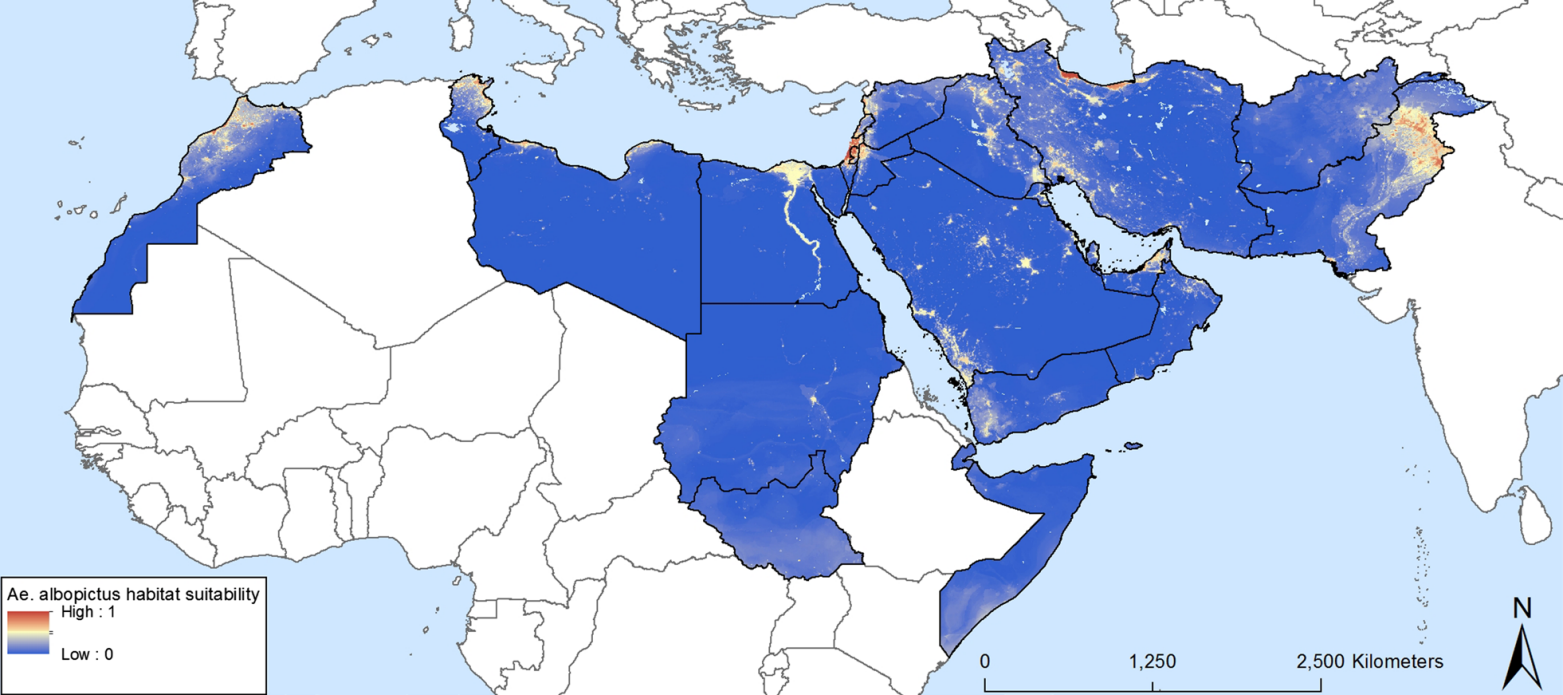
Predicted probability of *Ae. albopictus* occurrence obtained from a random forest model

**Fig. 8:**
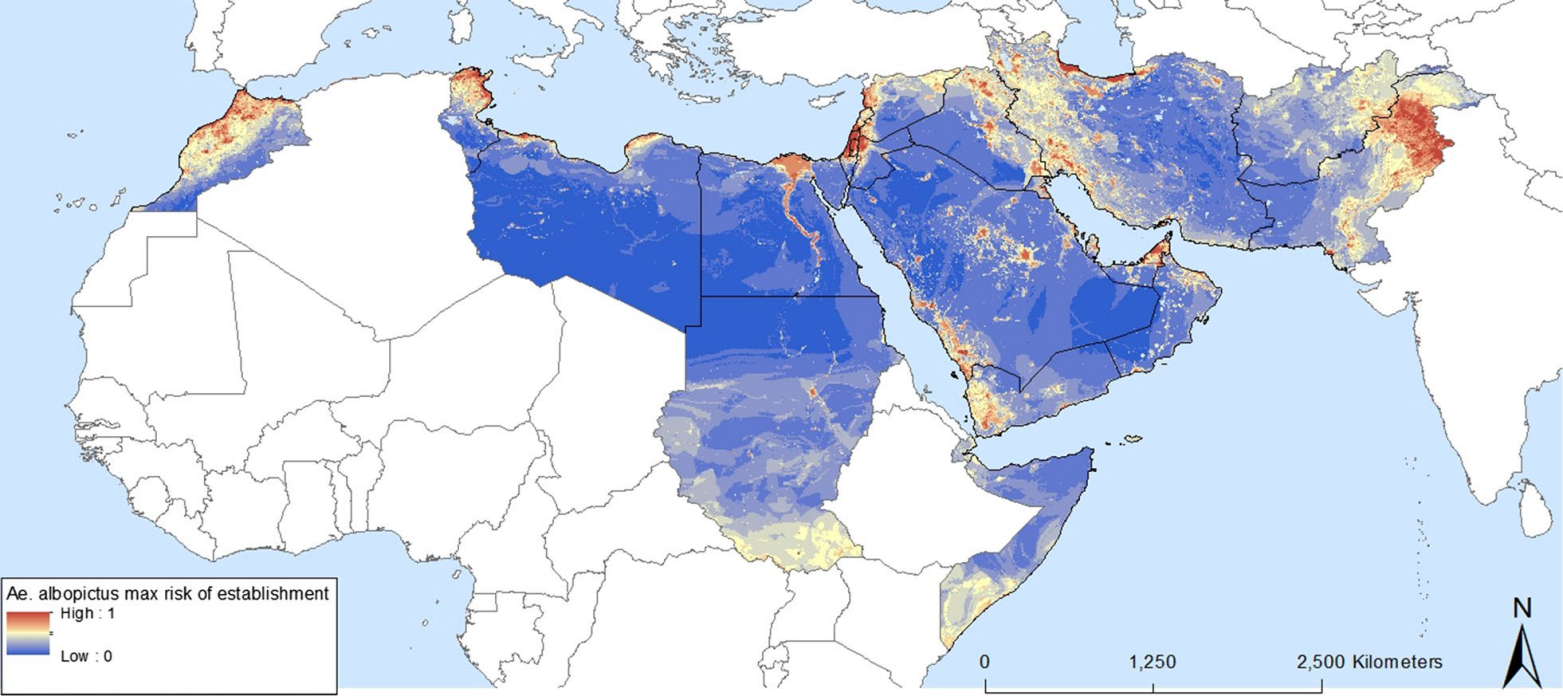
Predicted probability of *Ae. albopictus* occurrence using maximum values at the pixel level from a series of 100 random forest models

**Fig. 9:**
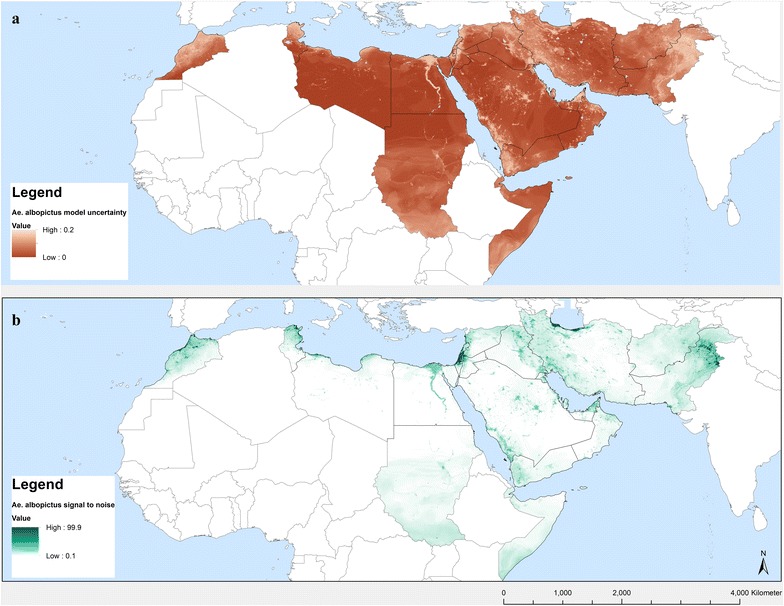
**a** Uncertainty of the *Ae. albopictus* predicted probability of occurrence using standard deviations at the pixel level from a series of 100 random forest models and **b** signal to noise ratio

The distribution model and maximum risk of establishments are shown in Figs. 4, 5, 6, 7, 8 and 9.

The predicted probabilities of establishment and spread of *Ae. aegypti* in the EMR are displayed in Figs. 4 and 5, respectively. The first map shows areas of high probability of occurrence, these being the southern areas of Somalia and Sudan, South Sudan (not part of EMR), the Nile delta in Egypt, the Red Sea border of Saudi Arabia and Yemen, and Pakistan. Areas suitable for maximum risk of establishment are highlighted in Fig. 5. These areas are Morocco, the Mediterranean Sea border of Tunisia, Libya and Egypt, Palestine, Lebanon, Syria and countries around the Red Sea rim and the Persian Gulf. This highlights that all EMR countries are suitable for *Ae. aegypti* establishment. The uncertainty map in Fig. 6a shows areas with a high standard deviation of model predictions. Among these are areas in Morocco and Oman and a band between Iraq and Iran that was not identified as being suitable in the distribution model. The signal to noise map (Fig. 6b) indicates that the noise is highest in the south of Sudan, which could be attributed to forested areas.

The predicted probabilities of *Ae. albopictus* occurrence in the EMR are shown in Figs. 7 and 8, which map areas of suitability and maximum risk of establishment, respectively. The maps highlight the fact that all EMR countries have areas suitable for the *Ae. albopictus*. They also confirm field observations in Morocco, Palestine, Jordan, Lebanon, Syria and Pakistan. Both maps show patchy zones of higher probability, corresponding to urbanized areas. Suitable regions with a high probability of establishment were also found in countries that, so far have not reported any occurrence such as Tunisia, Libya, Egypt, Iraq, United Arab Emirates, Saudi Arabia (south-western region), and Yemen. Figure 8 indicates where the maximum risk of establishment of *Ae. albopictus* could be found. This includes the Nile delta in Egypt, the southern zones of South Sudan (outside EMR) and Somalia, and eastern Afghanistan. A map of uncertainty associated with the predictions for *Ae. albopictus* is presented in Fig. 9a. In the case of *Ae. albopictus* the noise is highest at the Mediterranean coast.

**Fig. 10:**
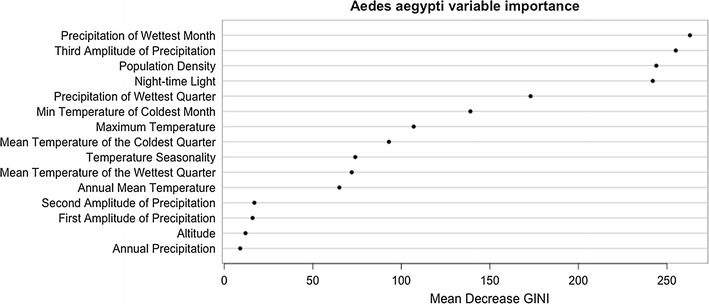
Dot chart of variable importance for predicting the occurrence of *Ae. aegypti*. Only the 15 most important variables are given

**Fig. 11:**
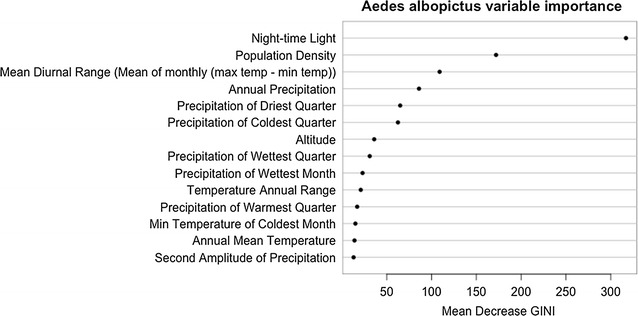
Dot chart of variable importance for predicting the occurrence of *Ae. albopictus*. Only the 15 most important variables are given

The random forest models show that the distributions of both *Aedes* species are highly influenced by demographic and climatic factors. Their relative importance is illustrated in Figs. 10 and 11 for *Ae. aegypti* and *Ae. albopictus*, respectively.

The five most important variables for *Ae. aegypti* were all related to precipitation and host availability, expressed as population density and night-time lights. The temperature variables scored less and ranked between rank five and rank ten. In case of *Aedes albopictus*, urbanisation was the most important factor, determined by population density and night-time lights. This is followed by temperature and precipitation.

### Internal and external validation

We received feedback from Tunisia, Oman, Pakistan and Somalia. Feedback from Somalia could not be quantified, as the points of contacts generally confirmed the findings but did not indicate any regions or disagreement. The accuracy indices of Tunisia, Oman and Pakistan are listed in Table 2. The accuracy expressed as Percent Correctly Classified is very high in all cases. Similarly, Cohen’s index of agreement ${\varvec{\upkappa}}$ can be classified as near perfect according to the benchmark categories defined by Landis and Koch [23]. The sensitivity and specificity measures indicate that there exists a good discrimination between the classes 0 (not suitable) on the one hand and the classes 1 and 2 (current suitability and maximum risk of establishment) on the other hand. Between the two classes there is confusion, mostly pixels that are considered maximum risk of establishment whereas they are currently suitable.

**Table 2:** Results of the country-based expert validation

Country	Accuracy (CI)	Kappa	Sensitivity			Specificity		
			0	1	2	0	1	2
Tunisia	0.989 (0.985, 0.994)	0.976	0.98	1	0.99	0.992	0.998	0.991
Oman	0.987 (0.982, 0.992)	0.968	1	0.561	1	1	1	0.936
Pakistan	0.928 (0.916, 0.939)	0.879	1	0.75	0.93	0.85	1	0.994

## Discussion

The distributions of *Ae. aegypti* and *Ae. albopictus* are, as expected, highly influenced by precipitation, demographic factors and temperature [24, 25]. Night-time light and human population density are among the most important predictor variables for both *Aedes* species. Night-time light indicate urbanisation and both species are container-breeders within an urban environment [26]. Additionally, as both species are anthropophilic, with *Ae. albopictus* being the most opportunistic [27], human population indicates host availability.

The relative importance of precipitation is highly pronounced for *Ae. aegypti*, for which the importance of precipitation amplitude 3 and the precipitation of the wettest month is comparable to that of demographic factors. In comparison to other studies [16, 17], the variable importance of precipitation variables seems higher. We must bear in mind that the EMR is an arid area with shortages in water supply. Therefore, precipitation may be more important than in other areas. These results highlight the fact that different key limit factors must be more relevant in different geographical areas, as suggested by Cunze et al. [28]. Altitude is included in the model in contrast to the study by Rochlin et al. [29] where this was irrelevant.

**Fig. 12:**
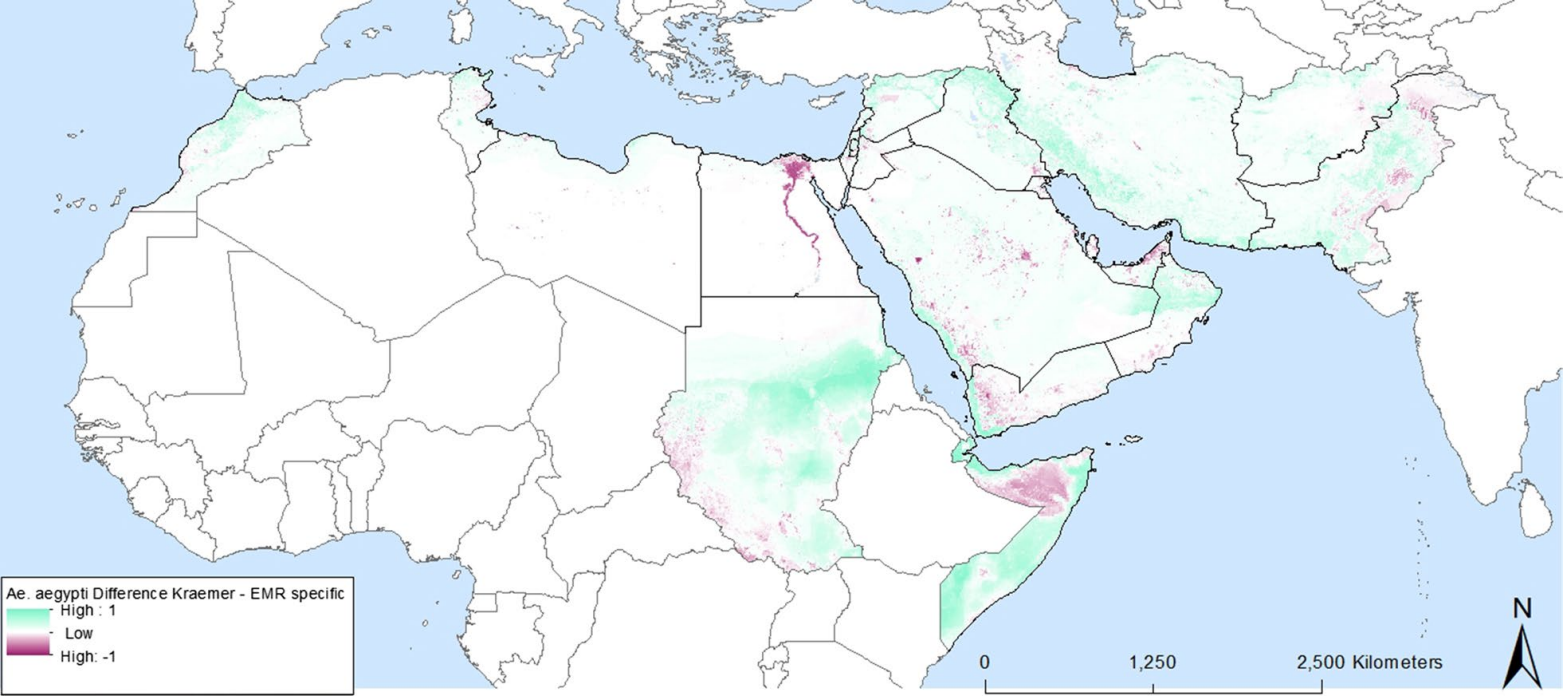
Map difference between Kraemer et al. [1, 2] and EMR specific *Ae. aegypti* probability

**Fig. 13:**
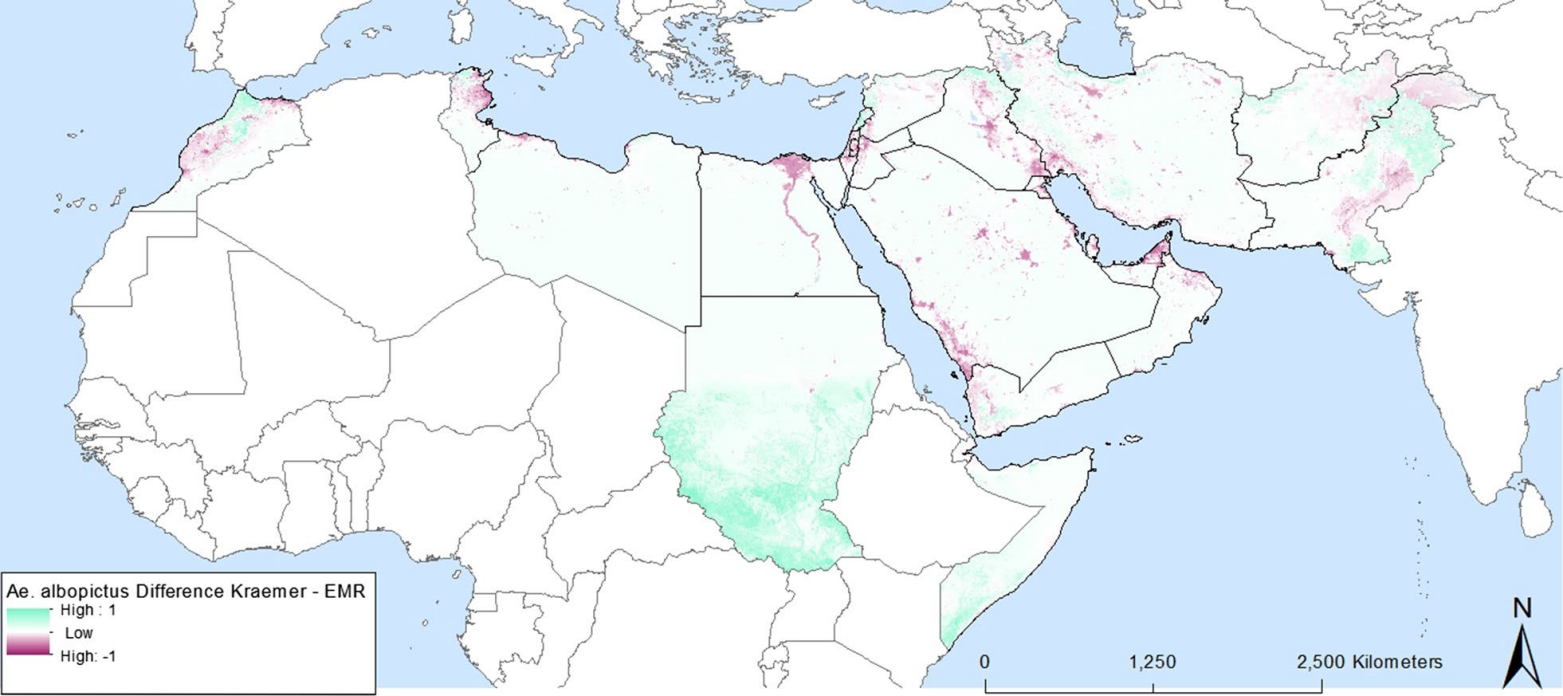
Map difference between Kraemer et al. [1, 2] and EMR specific *Ae. albopictus* probability

When the EMR specific model output is contrasted to the model output generated by Kraemer et al. [1] (Figs. 12, 13) it is clear that the models differ especially in urban areas within the EMR. Whilst this might be considered a small spatial difference, it has serious implications in terms of vector-borne disease management. Indeed, these areas are where the highest population density can be found so if vectors and/or pathogens are introduced within these high probability areas this might lead to outbreaks, as confirmed by the reported cases of dengue and chikungunya over the last years (Additional file 1). Other modelling approaches such as the MaxEnt output from Medley [16] even completely failed to highlight suitability within the EMR.

The results of the distribution and maximum risk of establishment models show that there are numerous areas with suitable habitats for *Ae. aegypti* and *Ae. albopictus* throughout the EMR, although few field data are available for the region. Monitoring these areas will help detecting introduction of the species in areas that are generally regarded as less suitable for the species. This also includes reintroduction, as *Ae. aegypti* was widely distributed in the Mediterranean during the last century [30].

We cannot exclude that these other areas within the EMR have already been invaded, as there is little information available on *Ae. aegypti* and *Ae. albopictus* in the EMR. Therefore, increasing *Aedes* and *Aedes*-borne disease entomological and epidemiological surveillance in the area is a priority. While there is an urgent need to undertake periodic surveillance campaigns in areas that are currently considered suitable for maximum risk of establishment, attention should also be paid to surveillance at larger population centres, at Points of Entry (PoE) for *Aedes*, such as harbours, roads and ground-crossings, and for *Aedes*-borne diseases and viruses, at airports and ports and larger urban areas.

As a first step towards capacity building for entomological surveillance in the area, training courses and guidelines for improving *Aedes* and *Aedes*-borne disease surveillance and control have been developed by the WHO Regional Office for the Eastern Mediterranean. However, it is also important to raise awareness of the key elements that affect habitat suitability for mosquitoes, especially in urban areas, such as unprotected storage of drinking water.

## Conclusion

This paper presented tailored distribution and maximum risk of establishment maps for the two major vectors of disease: *Aedes aegypti* and *Ae. albopictus* for the WHO EMR. Previous maps were highly biased towards data from the Americas and Asia and failed to identify risk areas in the target region. The obtained maps were provided to the points of contacts within the countries and their expertise was used to validate the outcome. Urban environment and host availability are among the most important predictor variables for both *Aedes* species. The relative importance of precipitation is especially pronounced for *Ae. aegypti* which reflects the aridity of the region with shortages in water supply.

The maps generated specifically for the EMR highlighted data gaps and these gaps will be filled using targeted monitoring and surveillance. This will increase the awareness and preparedness of the different countries for *Aedes* borne diseases.

## Additional files

**Additional file 1.** Presence of *Aedes aegypti* and *Aedes albopictus* and case data of Dengue, chikungunya and yellow fever in the EMR.

**Additional file 2: Fig. S1.** Presence data (*Ae. aegypti* and *Ae. albopictus*) taken from locations in areas with a Mahalanobis distance greater than 280 that were excluded from the model training data.

**Additional file 3: Fig. S2.** TFA processed precipitation data before (top) and after (bottom) spatial interpolation.
