# Supplementary figures and images for: Current and future distribution of Aedes aegypti and Aedes albopictus (Diptera: Culicidae) in WHO Eastern Mediterranean Region
Source: Int J Health Geogr. 2018 Feb 14;17:4. doi: 10.1186/s12942-018-0125-0 (PMC5813415; doi:10.1186/s12942-018-0125-0)

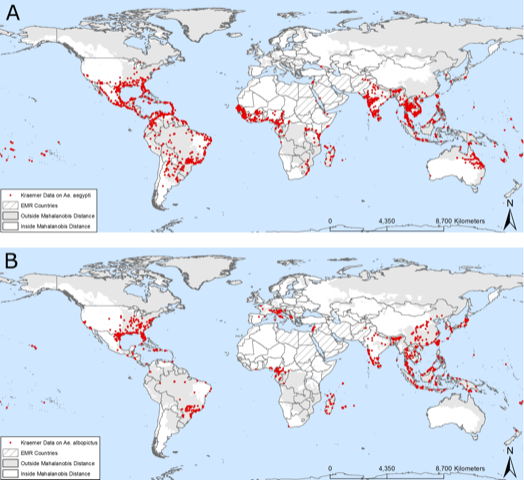

Supplement: Supplementary file 2 — Additional file 2: Fig. S1. Presence data (Ae. aegypti and Ae. albopictus) taken from locations in areas with a Mahalanobis distance greater than 280 that were excluded from the model training data. [file 12942_2018_125_MOESM2_ESM.docx]

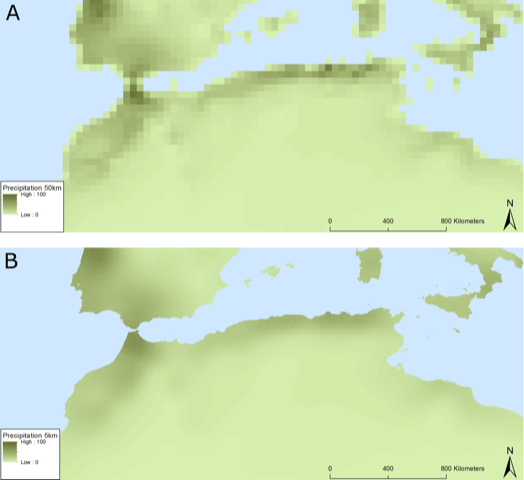

Supplement: Supplementary file 3 — Additional file 3: Fig. S2. TFA processed precipitation data before (top) and after (bottom) spatial interpolation. [file 12942_2018_125_MOESM3_ESM.docx]
